# A Special Issue on “Food Perception and Preferences in the Context of Health and Sustainability”

**DOI:** 10.3390/foods13091394

**Published:** 2024-05-01

**Authors:** Marlies Wallner, Monica Laureati

**Affiliations:** 1 Institute of Dietetics and Nutrition, University of Applied Sciences FH JOANNEUM, 8020 Graz, Austria; 2 Department of Food, Environmental and Nutritional Sciences, University of Milan, 20133 Milano, Italy; monica.laureati@unimi.it

In a rapidly changing world with increasing environmental and health issues, it is necessary to steer research in an appropriate direction while keeping our health in mind. Food choices have a profound impact on our planet, our environment, and our wellbeing. Food choices are mainly determined by food preferences, which in turn rely to a very large extent on individual perception. For this reason, considering consumer preferences in the development of affordable innovative health-promoting foods is extremely relevant and can contribute to the products’ success while fostering the transition towards more sustainable and healthy dietary patterns. Moreover, knowledge about sustainable nutrition is crucial to make adequate purchasing decisions and strategies to reach out to different target groups, which are of high relevance. It was therefore important for us to offer a platform for this within the framework of this Special Issue and to collect relevant studies.

In this Special Issue, research on healthy and sustainable products was published. Freschi and colleagues, for example, tested consumer acceptance of a sustainable version of salami using game meat from a wild boar. The production of local game meat is a proven way of promoting sustainable food, which is also consistent with the proper management of the expansion of the wild boar species since it is considered 1 of the 100 worst invasive animals responsible for the spread of pests. They showed that food enjoyment was influenced by the different spices used to mask the intense smell of wild board meat, rather than by the ratio of wild boar to pork. This finding suggests that it is possible to produce cost-effective and environmentally friendly products, as doughs with a high proportion of wild boar meat can be used without affecting product preference [1]. 

The use of pseudo grains, minor crops, and alternative protein sources in products is a key factor in the future of nutrition. Not only do they have a positive impact on the environment, but some of them are also necessary to fulfil the nutritional requirements of specific target groups. Buckwheat and maize are used in gluten-free products, which are relevant for coeliacs and have other health benefits. The sensory challenge is to integrate these often bitter and astringent yet health-promoting ingredients into a well-accepted product. The results of Rabitti et al. describe how the addition of up to 30% buckwheat flour, an interesting minor crop with an excellent nutritional profile that contributes to the sustainability and biodiversity of the agri-food system, can be used to obtain gluten-free food formulations with optimal sensory properties [2]. On the other hand, in the systematic review by Abreu et al., innovative approaches to integrate lupin into food products to improve their fiber and protein content were reported [3]. The review revealed a positive trend in consumers with attributes like taste, texture, and appearance playing crucial roles. Consumers appreciated lupin-based products as alternatives to traditional sources of protein, particularly in meat analogs and dairy substitutes.

In these regards, Wallner and colleagues developed and tested a pasta sauce containing mealworm, which has recently been approved as a novel food. The complete substitution of meat with mealworm was less accepted by consumers, but partial substitution in a common meat sauce was well accepted and therefore had potential as a future ingredient for our diets [4]. Another investigation dealt with the sensory properties of plant-based yogurts. Vanilla and lemon aromas were added to enhance the flavor profiles of dairy-free yogurts. Static sensory analysis involves assessing various attributes, such as taste, texture, and aroma, while dynamic methods consider how these attributes change over time. The findings suggest that the addition of vanilla and lemon aromas positively affects the overall sensory experience of plant-based yogurts, enhancing taste and aroma perceptions. Dynamic sensory analysis reveals that these aromas can have a sustained impact on flavor perception throughout consumption, making them valuable additions for improving the palatability of plant-based yogurt products [5].

Furthermore, a study by Collier et al. delved into the psychological and practical aspects of reducing and substituting meat to make more sustainable food choices. Researchers explored the factors that influence individuals’ decisions to cut down on meat consumption and opt for alternatives. They found that both psychological factors, such as attitudes and beliefs about meat, and practical considerations, such as the availability of meat substitutes, played significant roles in shaping dietary choices. The study highlighted the importance of addressing both psychological and practical aspects to encourage more sustainable food choices, emphasizing that making such choices can have a positive impact on the environment [6]. 

In addition to the development of new and future products, the perception of food and behavioral influences are an important research area to better understand consumers’ choices. In a study by Winzer et al., the association between taste preference, sugar-sweetened beverage consumption, and anthropometric parameters was investigated in preadolescents. The findings revealed that pre-adolescents with a preference for sweet taste tended to consume more sugary drinks. The study highlighted the importance of considering taste preferences when designing interventions to promote healthier beverage choices. These results highlight the importance of taste as explanatory variable in eating behaviors and can be relevant to develop strategies for reducing the consumption of sugar-sweetened beverages in this target group [7]. 

Research into the effectiveness of measures for health and the environment is important for the understanding of the influences on weight management as well as for the sustainable use of food regarding food waste. In this regard, the role of tableware size was tested by Abeywickrema et al. in influencing healthy eating behaviors, with its effects on subsequent food intake particularly playing a role in overeating and therefore in the development of overweight. Researchers conducted experiments to determine how the size of plates and bowls affected the amount of food consumed by individuals. The findings indicated that small tableware led to increased post-meal satiety and reduced post-breakfast energy intake. However, within the following meal, this effect was counteracted by a higher energy uptake. This finding suggests that small tableware size might not have a beneficial long-term effect on reducing energy uptake [8]. 

Furthermore, another research group aimed to evaluate the effectiveness of health- and environment-focused text messages in promoting the consumption of a sustainable diet among young adults, with a specific focus on the importance of expected taste. Researchers conducted experiments involving text message interventions to encourage sustainable food choices. The findings indicated that messages emphasizing expected taste played a significant role in increasing the consumption of sustainable foods among young adults. This study highlighted the potential of tailored messaging strategies that consider taste preferences to promote more sustainable dietary choices in this demographic [9].

Another study aimed to investigate the factors influencing consumers’ intentions to repurchase organic food grain, with a focus on sustainable consumption. Researchers conducted surveys and analyzed data to identify key determinants of repurchase intentions among consumers. The findings revealed that several significant factors, including perceived product quality, environmental concern, and personal values were related to sustainability. The study highlighted the importance of these factors in influencing consumers’ decisions to continue purchasing organic food grain, emphasizing the role of sustainability considerations in shaping purchasing behaviors [10].

In addition to research on individuals, the involvement of the entire food system, including relevant stakeholders, is important to derive meaningful recommendations for action. Lastly, Balan et al. explored the concept of metabolic food waste as a contributing factor regarding food insecurity, focusing on its causes and prevention. Researchers conducted an analysis to identify the underlying factors leading to metabolic food waste in food systems. They highlighted issues such as overproduction, improper storage, and consumer food waste as significant contributors. The study emphasized the importance of proactive measures and policy interventions to reduce metabolic food waste as a means to address food insecurity and promote more efficient and sustainable food systems [11]. 

Moreover, we would like to thank the researchers who published their research within this Special Issie; we are pleased to have compiled a relevant collection of studies relating to sensory science, health, and sustainability.
